# Evaluation of Alternative Euthanasia Methods of Neonatal Chickens

**DOI:** 10.3390/ani8030037

**Published:** 2018-03-09

**Authors:** Shailesh Gurung, Dima White, Gregory Archer, Dan Zhao, Yuhua Farnell, J. Allen Byrd, E. David Peebles, Morgan Farnell

**Affiliations:** 1 Department of Poultry Science, Texas A&M AgriLife Research, College Station, TX 77843, USA; sg45902@tamu.edu (S.G.); dima.white@vikings.berry.edu (D.W.); garcher@tamu.edu (G.A.); dz137@tamu.edu (D.Z.); yfarnell@tamu.edu (Y.F.); 2 Southern Plains Agricultural Research Center, Agricultural Research Service, US Department of Agriculture, College Station, TX 77843, USA; byrdmen8@yahoo.com; 3 Department of Poultry Science, Mississippi State University, Mississippi State, MS 39762, USA; d.peebles@msstate.edu

**Keywords:** euthanasia, gas inhalation, low atmospheric pressure stunning, male layer chicks

## Abstract

**Simple Summary:**

Male layer chicks do not have economic value and are humanely killed after hatching. The layer industry is seeking alternative methods to humanely kill recently hatched male chicks. This study evaluated the use of gases or negative air pressure as a means of humane and viable alternatives to maceration. The treatments included carbon dioxide, nitrogen, reduced air pressure, and a negative control. The study showed that chicks exposed to treatments, gases or negative air pressure, did not differ significantly in terms of the physiological stress response. The use of carbon dioxide resulted in a faster onset of unconsciousness and ultimately death as compared to nitrogen or negative air pressure treatments.

**Abstract:**

Hatched male layer chicks are currently euthanized by maceration in the United States. Public concerns on the use of maceration have led to the search for alternative methods. We hypothesized that gas inhalation and low atmospheric pressure stunning (LAPS) are viable and humane alternatives to instantaneous mechanical destruction. The objective of this study was to evaluate the physiological and behavioral responses of recently hatched male layer chicks when subjected to carbon dioxide, nitrogen inhalation, or LAPS. The study consisted of seven treatments: breathing air (NEG), 25% carbon dioxide (CO_2_), 50% CO_2_, 75% CO_2_, 90% CO_2_, 100% nitrogen (N_2_), or LAPS. Ten day-of-hatch, male layer chicks were randomly assigned to each treatment, and each treatment was replicated on ten different days. A custom-made vacuum system was used to reduce air pressure inside the chamber from 100.12 kPa to 15.3 kPa for the LAPS treatment. Serum corticosterone and serotonin levels were measured using commercially available competitive enzyme linked immunosorbent assay (ELISA). Latencies to loss of posture and motionlessness were determined from video recordings. The 25% and 50% CO_2_ treatments were discontinued after the first replication, as the majority of the chicks recovered. The chicks in the negative (NEG) group had significantly higher levels of corticosterone than the other four euthanasia treatments. On the other hand, the serotonin levels of chicks in the NEG group was significantly lower when compared to the other four euthanasia treatments. The latencies to loss of posture and motionlessness of chicks exposed to 75% and 90% CO_2_ were significantly shorter than those in the LAPS and N_2_ inhalation treatments. These data suggest that the stress responses of chicks to the CO_2_, N_2_, and LAPS treatments do not differ among each other. However, the CO_2_ inhalation method was faster in inducing loss of posture and motionlessness in chicks than the LAPS and N_2_ inhalation treatments.

## 1. Introduction

Genetic selection of chickens for either egg or meat production has benefitted the poultry industry and its consumers. The broiler industry raises both males and females for meat production, while the layer industry rears females only for egg output. The global egg industry was comprised of 7.3 billion hens in 2015, which produced 70.8 million metric tons of eggs [1]. In the US alone, 365 million laying hens produced 88.4 billion table eggs in 2016 [2]. For each laying hen that is hatched, a male chick will also be produced. However, male layers do not have any economic value. These birds do not produce eggs, grow slowly, have a poor feed conversion ratio, incur high fattening costs to producers, and yield inferior quality meat [3,4]. Therefore, recently hatched male layer chicks are euthanized after being sexed in hatcheries. In the European Union (EU), that number is approximately 280 million chicks per year [5].

The American Veterinary Medical Association (AVMA) has approved maceration and carbon dioxide (CO_2_) gassing as acceptable methods for euthanasia of chicks up to 72 h of age [6]. Maceration results in the instantaneous death of chicks, poults, and embryonated eggs by physical disruption of the brain [6]. The method requires special equipment, called macerators, which have rotating blades for rapid fragmentation and death. Carbon dioxide inhalation quickly reduces intracellular pH, resulting in respiratory acidosis and anesthesia [7]. Continued exposure to the gas leads to hypercapnic hypoxia, respiratory depression, and ultimately death [8]. Raj and Whittington [9] studied the efficacy of either a 90% CO_2_ treatment or mixture of 20, 30, 40% CO_2_ in argon. Their study [9] reported that day-old chicks subjected to 90% CO_2_ in the air died within two minutes of exposure. European Union guidelines of Council Directive 93/119/EC on animal protection at the time of slaughter or killing, requires chicks to be exposed to the highest possible concentration of CO_2_ dispensed from a 100% source and requires that the gas concentration be maintained until the death of the chicks [10]. Nitrogen (N_2_) and argon (Ar) are tasteless, odorless gases that displace oxygen from breathing air, resulting in a loss of consciousness from anoxia, which finally leads to bird death [11]. Residual oxygen concentration should be maintained below 2% while using these anoxic gases for euthanasia [9,12]. Raj and Whittington [9] reported that the recovery rates of chicks exposed to mixtures of 20 to 40% CO_2_ in Ar, with a 5% residual oxygen concentration, ranged from 15 to 100%.

An alternative method for poultry stunning is by the reduction in atmospheric pressure [13]. The method is known as low atmospheric pressure stunning (LAPS) [14,15]. This process induces unconsciousness in subjects by decreasing air pressure inside a chamber, subsequently decreasing partial oxygen pressure and ultimately causing hypoxia [14,16,17]. The LAPS method received a ‘no objection’ status from the United States Department of Agriculture (USDA) in 2010 [13,17]. Purswell et al. [15] stated that during LAPS, the anatomy of the avian respiratory system makes it unlikely for gases to be trapped in the body cavities of chickens, unless the trachea is blocked. Additionally, newly hatched male chicks do not have access to feed and have minimal microbial gut colonization, making potential buildup of gases in the gut unlikely.

Methods of humane killing such as gas inhalation, maceration, and LAPS have led to welfare concerns by the public. Inhalation of CO_2_ gas at concentrations between 40% and 55% can cause painful sensations in humans due to acidification of the nasal mucosa [18]. Chickens have arterial and central chemoreceptors that respond to CO_2_ upon inhalation. These birds are likely to experience distress when exposed to high CO_2_ levels [12]. Hens exposed to CO_2_ gas concentrations of 40 to 50% may experience pain or discomfort [19]. Raj et al. [20] reported that a 47% CO_2_ gas composition in air is aversive to laying hens. Poultry subjected to CO_2_ demonstrate signs of respiratory discomfort such as sneezing, gasping and head shaking [21]. Chicken embryos are exposed to CO_2_ concentrations as high as 14% [22] and, therefore, may require higher concentrations of CO_2_ and longer exposure times to be euthanized when compared to adult chickens [6]. However, birds do not exhibit aversive behavior when initially exposed to inert gases [11,12]. Sandilands et al. [23] reported that broilers found the three gas mixtures—50% CO_2_ in air, 90% N_2_ in CO_2_, and 80% Argon in CO_2_—aversive but, comparatively, inert gas mixtures induced fewer signs of respiratory discomfort than CO_2_ in air. Nevertheless, birds subjected to inert gases exhibit severe wing flapping and convulsions [16]. Exposure to air pressure that has been reduced to 17.8 kPa for two minutes has been shown to be lethal to broilers [15]. However, the requirement for day-old chicks and poults may be different. Therefore, scientific studies concerning potential applications of LAPS for euthanizing recently hatched chicks and poults are needed.

Scientific studies that have evaluated the current euthanasia procedures for day-old chicks are limited [24]. Raj and Whittington’s [9] paper entitled “Euthanasia of day-old chicks with carbon dioxide and argon” is the single research article found in the scientific record that addresses day-old chick euthanasia. To the authors’ knowledge, studies on behavioral and physiological responses of day-old male layers to these different euthanasia methods have not yet been published in the scientific literature. The poultry industry is also looking for humane and viable alternative methods to replace maceration of neonatal chickens. The objective of this study was to develop other viable alternative methods for chick euthanasia. The specific objectives were to evaluate physiological responses of male layer neonates to gas inhalation and LAPS by measuring serum corticosterone (CORT) and serotonin (5-HT) concentrations, and to assess behavioral responses of chicks such as latencies to loss of posture and motionlessness.

## 2. Materials and Methods

### 2.1. Experimental Design

Alternative methods to maceration were evaluated and compared in this study. Along with a negative control (Air), experimental treatments included 25% CO_2_, 50% CO_2_, 75% CO_2_, 90% CO_2_, 100% N_2_, and LAPS. Since it was practically unfeasible to achieve a 100% CO_2_ concentration inside the chamber, a 90% CO_2_ treatment was used as one of the treatments. Chicks were subjected to air as the negative control treatment. This treatment was used to assess potential effects of the experimental chamber, handling process, and flow of gases on the experimental birds. In each treatment, a batch of 10 day-of-hatch Hy-Line W-36 male chicks were used. Each treatment was replicated on 10 different days. The 25% CO_2_ and 50% CO_2_ treatments were discontinued after the first replicate day, due to a high number of survivors. A total of 520 male layer chicks were used during the entire study. The chicks were provided with clean drinking water, and a 250 Watt heating lamp was used to maintain optimal temperature during the trial. All birds were cared for under an approved Texas A&M University Institutional Animal Care and Use Committee protocol (IACUC 2016-0200).

### 2.2. Experimental Chamber

A custom-built vacuum system mounted on a cart was procured from a manufacturer (LACO Technologies, Salt Lake City, UT, USA). It included a horizontal cylindrical vacuum chamber, with an internal diameter of 0.45 m, length of 0.5 m, and internal volume of 80 L. The system had a clear acrylic lid, a vacuum pump of flow rate of 201 L/m (7.1 cfm) and a vacuum controller unit. The vacuum chamber was also used as the experimental chamber for all seven treatments. A thermocouple measured the temperature of the chamber during each treatment. The temperature of the chamber did not change during application of any of the gases—CO_2_, N_2_, and air, as well as during LAPS. It was unlikely that the chicks experienced any cold stress, since the temperature remained within a fairly constant range of 20.5 °C to 23.3 °C between treatments.

### 2.3. Experimental Procedure

**Figure 1:**
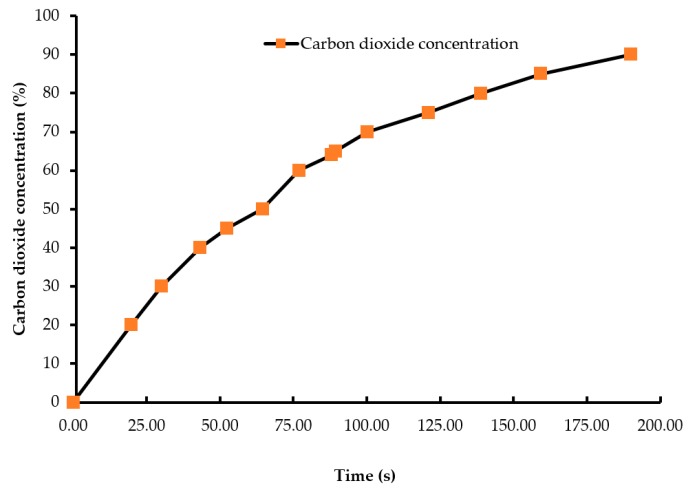
Carbon dioxide wash-in curve (concentration vs. time). The carbon dioxide concentration in the beginning of the treatment was 0.04% which increased to 90% as the chamber was being filled by carbon dioxide gas. An infrared CO_2_ sensor measured the gas concentration inside the chamber during the experiment.

**Figure 2:**
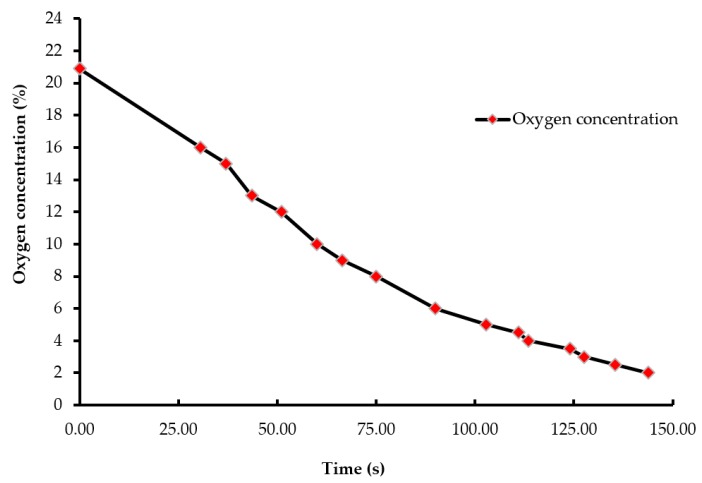
Oxygen wash-out curve (concentration vs. time). The oxygen concentration at the beginning of the treatment was 20.9%, which decreased to less than 2% as the chamber was being filled by nitrogen gas. An electrochemical oxygen sensor measured the oxygen concentration inside the chamber.

**Figure 3:**
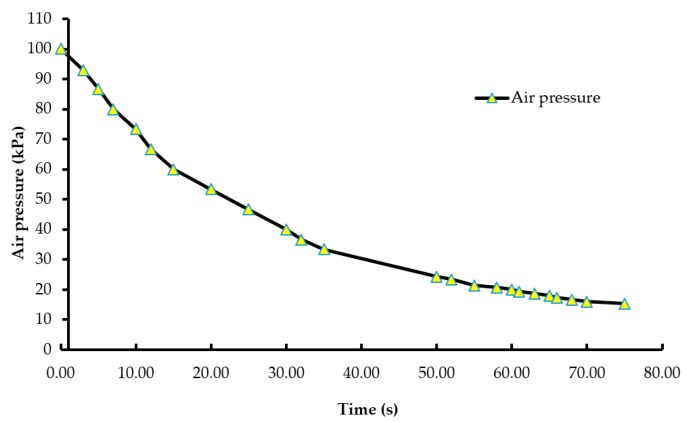
Pressure wash-out curve (pressure vs. time). The air pressure of the chamber was decreased from 100.12 kPa to 15.3 kPa during the LAPS treatment. The custom-built vacuum system was equipped with a vacuum controller, which measured and maintained negative air pressure within the chamber.

A batch of ten chicks was exposed to each treatment gas or a reduction in air pressure until the desired concentration or final air pressure was achieved inside the chamber. The chicks were then held inside the chamber for an additional five minutes. Gas tanks containing 100% CO_2_, 100% N_2_, and breathing air were procured from a local supplier for the study. A uniform gas delivery pressure of 103.4 kPa was used in all gas treatments. A variable area flow meter (Cole-Parmer, Vernon Hills, IL, USA) was used to control the flow rates of the gases into the chamber. Flow rates of all CO_2_ treatments were set at 42 L/m, while that of the 100% N_2_ and negative control (air) treatments were set at 60 L/m. The time taken to achieve 90% CO_2_ in the CO_2_ treatment was 195 s, and 2% O_2_ took 144 s in the N_2_ treatment (Figure 1 and Figure 2). However, in the N_2_ treatment, gas was allowed to fill the chamber for a total time of 195 s. A higher flow rate was used in the N_2_ treatment to achieve a 100% concentration of gas in a similar amount of time as the other treatments. The negative control flow rate of breathing air was set at the same flow rate as the N_2_ treatment, for later comparisons. Carbon dioxide (100%) was delivered to the chicks until the desired concentration of CO_2_ was achieved inside the chamber. An infrared CO_2_ sensor (Servomex, Crowborough, UK) was used to measure and confirm the gas concentration. Nitrogen gas flowed into the chamber until the oxygen concentration was reduced to less than 2%. The oxygen concentration inside the chamber was measured using an electrochemical sensor (Sper Scientific, Tucson, AZ, USA). Figure 2 shows the oxygen washout process as nitrogen was filling the chamber. In the LAPS treatment, the air pressure inside the chamber was decreased gradually, which subsequently reduced the partial pressure of oxygen. Purswell et al. [15] estimated that a negative atmospheric pressure of 19.4 kPa would result in 99.9% broiler mortality. However, it was found in our laboratory that chicks survived the negative air pressures of 17.9 kPa and 19.4 kPa. Further preliminary trials determined that a negative air pressure of 15.3 kPa would result in 100% chick mortality. Therefore, air pressure inside the chamber was reduced from 100.12 kPa to 15.3 kPa in the LAPS treatment. The decrease in air pressure over time is shown in Figure 3. Once the required gas concentration or negative air pressure was achieved, chicks were exposed further for a period of 5 min. Chicks were observed for any signs of recovery after the end of that 5 min holding time.

### 2.4. Stress Physiology

Blood samples were collected from each chick by cardiac puncture after death had been verified in all treatments, except for those belonging to the negative control group. In the negative control group, chicks were decapitated within 30 s of their removal from the experimental chamber at the end of the duration of treatment and blood samples were collected subsequently. The blood samples were allowed to clot overnight at 4 °C. The next day, serum was collected from the blood samples by centrifuging at 500× *g* at 4 °C. Competitive enzyme linked immunosorbent assay (ELISA) kits ADI-901-097 and ADI-900-175, were used to measure the concentration of CORT and 5-HT, respectively (Enzo Life Sciences, Farmingdale, NY, USA), following the instructions in the kit manuals. All samples were run in duplicate. Intra-assay and inter-assay variabilities were 1.9% and 7.2%, respectively, for the corticosterone assay. Intra-assay and inter-assay variabilities for the 5-HT assay were 1.6% and 5.8%, respectively. Serum CORT and 5-HT concentrations were interpolated from a standard curve using curve-fitting software (Gen 5.0, Bio-Tek Instruments, Winooski, VT, USA).

### 2.5. Behavioral Observations

Treatments were videotaped for evaluation of behavioral responses of the chicks using a digital video camera (Sony, New York City, NY, USA). The variables measured were latencies to loss of posture and motionlessness. Each video segment included all the events starting from application of treatment till the end of the five min holding period. The time point when 100% of the chicks demonstrated the defined behavior was noted and the differences from the start of treatment were recorded as latency measurements. Chicks in all treatments, except in the air group, demonstrated ataxia, loss of posture, convulsions and motionlessness. Behaviors such as failure to maintain body balance, swaying of body, inability to stand, and flipping were signs of ataxia. The state of loss of posture was determined once the chicks were lying with necks extended and unable to control body posture [17,25]. This was followed by onset of convulsions in which signs like wing flapping, muscle twitching, leg paddling, and flipping over could be seen. These behavioral signs ended with straightening of legs and cessation of convulsions. The state of motionlessness was established once any visible movements including breathing motion had stopped [17,25]. Latencies to loss of posture and motionlessness were recorded as time durations after which the respective behaviors were exhibited. Other behaviors such as alertness, headshaking, gasping, neck extension and vocalizations were noticed but not quantified.

### 2.6. Statistical Analysis

The serum CORT and 5-HT levels obtained after ELISA and the data of behavioral variables were summarized in a spreadsheet (MS-EXCEL, Microsoft, Redmond, WA, USA). Data were statistically analyzed using a one-way analysis of variance following PROC ANOVA procedures (SAS 9.4, Cary, NC, USA). Least-squares means were compared using Fisher’s Least significant difference (LSD) post hoc test in the event of significant global effects [26]. The statistical tests were carried out at 5% significance level. Two CORT samples from the 90% CO_2_ treatment and one from 100% N_2_ treatment were identified as outliers by Tukey’s boxplot method and removed from study.

## 3. Results and Discussion

Day-of-hatch male chicks exposed to the 25% CO_2_ and 50% CO_2_ treatments recovered after the end of a five min holding period. All ten chicks in the 25% CO_2_ group recovered, and only two out of ten chicks died in the 50% CO_2_ group. Therefore, the two treatments were discontinued after the first replicate time period. Chicks exposed to a CO_2_ concentration of 75% or above for a period of five min did not recover. This finding demonstrates that chicks require a higher concentration of CO_2_ than adult birds. The results described are of the following five treatments: negative control (air), 75% CO_2_, 90% CO_2_, 100% N_2_, and LAPS. In the 75% and 90% CO_2_ treatments, air inside the chamber was displaced by CO_2_. Figure 1 demonstrates the wash-in curve for both CO_2_ treatments. A 100% N_2_ source was used to fill the chamber with this anoxic gas during N_2_ treatment. As N_2_ was being filled in the chamber oxygen levels continuously decreased. Figure 2 illustrates the wash-out process of oxygen during N_2_ treatment. Air pressure inside the chamber was gradually decreased during the LAPS treatment. Figure 3 shows the time taken for the reduction in air pressure of the chamber from 100.12 kPa to 15.3 kPa. At such a reduced air pressure level, oxygen fails to diffuse from alveoli into the capillaries because of decrease in partial oxygen pressure in the inspired air. This leads to death from anoxia.

### 3.1. Serum Corticosterone

**Figure 4:**
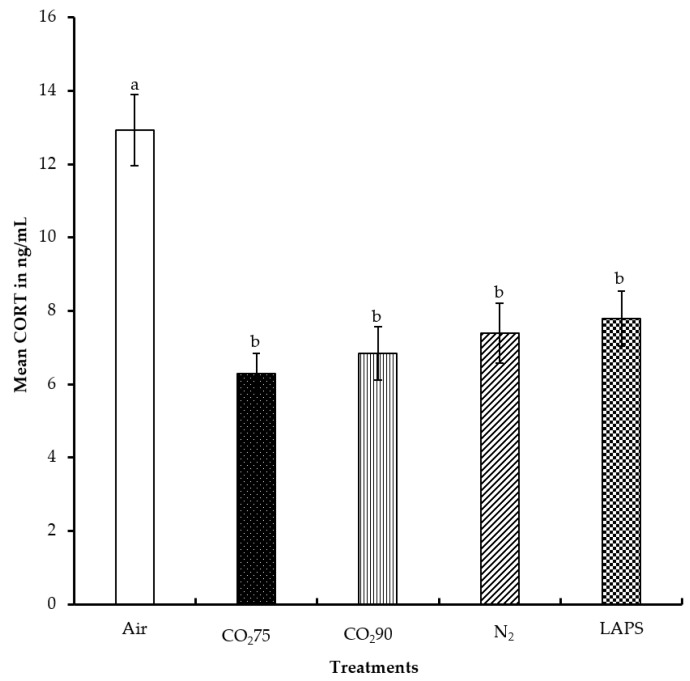
Mean serum corticosterone levels of male layer chicks. The CORT concentrations were measured in duplicate and expressed in ng/mL. Bars (mean ± SEM) with different superscripts (a,b) are significantly different by Fisher’s LSD test (*p* < 0.05). Number of samples per treatment was 100.

The average CORT levels of the chicks subjected to the negative control, 75% CO_2_, 90% CO_2_, 100% N_2_ and LAPS treatments were 12.9, 6.3, 6.8, 7.4, and 7.8 ng/mL, respectively (Figure 4). The chicks in the negative control treatment had a significantly higher serum CORT concentration than those chicks in all the rest of the treatments (*p* ≤ 0.0001). However, the serum CORT concentrations of the chicks subjected to the gas inhalation methods, namely 75% CO_2_, 90% CO_2_, and 100% N_2_, were similar to those of the LAPS treatment (*p* > 0.05). The chicks in the negative control group were alive for the longest duration among all the treatments, until they were decapitated for blood collection at the end of their treatment. The chamber was a novel environment for the chicks and the handling of chicks prior to decapitation for blood collection are probable reasons for their higher CORT levels.

Carbon dioxide causes pain and discomfort to the animal due to the formation of carbonic acid in the mucous membrane [27]. Kaye et al. [28] reported that hypercapnia due to CO_2_ stimulated the sympathetic nervous system and the hypothalamo-pituitary-adrenal axis. In the current study, the chicks belonging to 75% and 90% CO_2_ treatment groups might have lost consciousness before a potentially painful CO_2_ level was attained inside the chamber due to the analgesic [29] and anesthetic effect [30] of carbon dioxide gas. Therefore, the serum CORT levels were statistically lower as compared to those in the negative control group. Exposure to 100% N_2_ did not elevate CORT levels in the chicks in comparison to those chicks in the negative control treatment group. Birds lack intrapulmonary chemoreceptors sensitive to N_2_ [12] and do not demonstrate aversive responses in the beginning. In addition, normal breathing air consists of 78% N_2_ by volume. Hence, animals are continuously being exposed to higher levels of N_2_. In the LAPS treatment group, the chicks experienced a reduction in air pressure and a subsequent reduction in the partial pressure of oxygen. This led to a reduction in the diffusion of oxygen into the blood stream, causing the chicks to die from anoxia. The mechanism of death from the LAPS treatment is similar to that of gas inhalation methods. The CORT data also suggests that the LAPS method is similar to CO_2_ and N_2_ inhalation, in terms of a stress response. In a study by Vizzier-Thaxton et al. [14], broilers of market age and live weight ranging from 2.7 kg to 4.1 kg subjected to LAPS had significantly lower CORT levels as compared to electrical stunning. Shackling during electrical stunning might be the reason for the higher CORT concentrations.

### 3.2. Serum Serotonin

**Figure 5:**
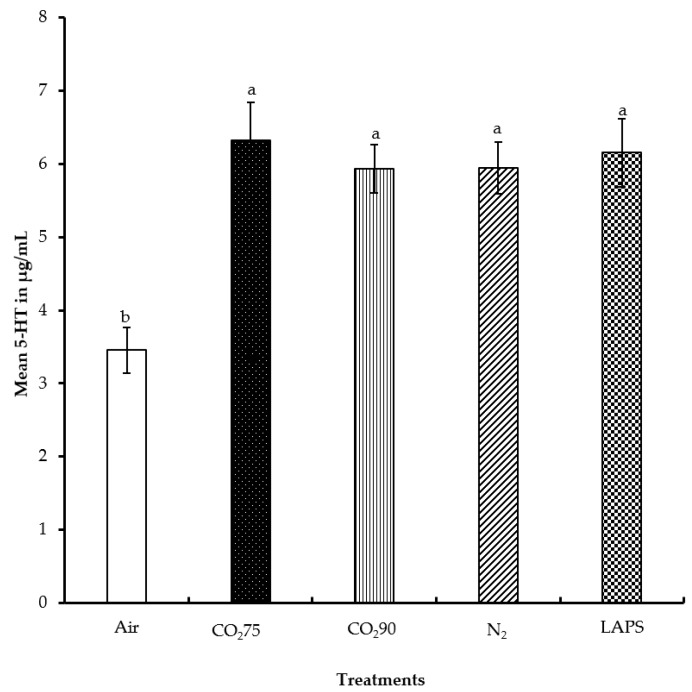
Mean serum serotonin levels of male layer chicks. The 5-HT concentrations were measured in duplicate and expressed in µg/mL. Bars (mean ± SEM) with different superscripts are significantly different by Fisher’s LSD test (*p* < 0.05). Number of samples per treatment was 20.

The 5-HT concentration in the peripheral blood of male layer chicks subjected to the five treatments were evaluated and compared. The mean serum 5-HT levels of chicks in the negative control, 75% CO_2_, 90% CO_2_, 100% N_2_, and LAPS treatments were 3.5, 6.3, 5.9, 5.9, and 6.2 µg/mL, respectively (Figure 5). Chicks in the negative control group had a significantly lower concentration of serum 5-HT than those in the rest of the treatments (*p* ≤ 0.0001). No significant differences were found in the 5-HT levels among the 75% CO_2_, 90% CO_2_, 100% N_2_, and LAPS treatment groups.

Serotonin has multiple functions in the central nervous system and in various peripheral tissues and organ systems. The level of 5-HT in the brain affects behavioral and neuropsychological processes such as mood, perception, memory, anger, aggression, fear, stress responses, appetite, behavior, and circadian rhythm [31]. Peripherally, 5-HT is vital in platelet aggregation, vasoconstriction, vasodilation, and intestinal motility [32]. In humans, anxiety disorders are related to a decrease in brain 5-HT levels [33]. Patients with depression have reduced whole blood [34,35] and platelet [36] 5-HT levels. Williams et al. [37] reported that higher blood 5-HT levels were positively associated with a better “mood”, in humans. Uitdehaag et al. [38] reported a moderate positive correlation between whole blood 5-HT and brain 5-HT of laying hens. The study suggested that peripheral 5-HT measurement might reflect brain 5-HT functioning. Rodenburg et al. [39] evaluated the effect of brooding and group selection for lower mortality on peripheral 5-HT serotonin and CORT levels of laying hens. The hens from randomly selected group raised without maternal care had a lower level of whole blood 5-HT as compared to birds from lower mortality group. Bolhuis et al. [40] studied the effect of selection against mortality on whole blood 5-HT and fear-related behavioral responses in beak trimmed or non-beak trimmed laying hens. The study observed that changes in peripheral 5-HT might be a feature of laying hens likely to demonstrate feather pecking behavior. In the present study, chicks in the negative control group had significantly higher CORT levels but lower 5-HT levels. Studies conducted in our laboratory on layer hen depopulation produced similar findings. Spent hens with higher CORT levels were found to have lower 5-HT levels [41]. A study by Inoue and Koyama [42] reported a decrease in 5-HT levels in the hippocampus of rats in response to acute corticosterone administration. In the present study, chicks in the 75% CO_2_, 90% CO_2_, 100% N_2_, and LAPS treatments had significantly higher 5-HT, but lower CORT levels as compared to those in the negative control group. The interesting relationship between serum CORT and 5-HT level needs further investigation.

However, exposure to CO_2_ results in hypercapnia increased CO_2_ tension in the blood, which is a stimulus for breathlessness [43]. Low atmospheric pressure stunning and nitrogen inhalation treatments act by reducing oxygen tension in the chamber and subsequently decreasing the O_2_ tension in blood. The ventilatory drive is increased immediately in case of CO_2_ inhalation and animals exhibit breathlessness. The experience of breathlessness has various degrees of unpleasantness and hence has potential to challenge animal welfare [43]. No assessments were made in this study among the treatments in terms of breathlessness.

### 3.3. Behavioral Responses

The behavioral responses of the chicks subjected to gas inhalation and LAPS treatments were video recorded. Chicks subjected to CO_2_ treatments demonstrated headshaking, gasping with neck extended immediately (approximately 10–15 s) after introducing the gas into the chamber. Headshakes, gasping and neck extension were more pronounced in chicks subjected to CO_2_ treatments (almost 100%) than in chicks subjected to N_2_ and LAPS. These are signs of aversion and have been reported in other studies [11,12]. These chicks then demonstrated signs of ataxia and loss of posture. Most of the chicks were lying in a prone position with slow and heavy open mouth breathing. Chicks subjected to 100% N_2_ and LAPS treatments showed a delayed response to gas introduction compared to chicks in the CO_2_ treatment. The prominent signs shown by chicks prior to loss of posture were similar in the N_2_ and LAPS treatments. Fewer of the chicks (1–2%) exhibited headshakes and gasping. These chicks looked drowsy, were falling on their sides and gradually shifting to sitting or prone position, and ultimately lost body posture and balance. Frequent labored or heavy breathing was seen in the chicks after losing posture. This was followed by convulsive movements such as wing flapping, leg paddling, and flipping over and was more common in chicks subjected to N_2_ and LAPS treatments (almost 100%). The period of latency until all chicks exhibited loss of posture or motionlessness were determined for all treatment groups, except for the negative control group.

**Figure 6:**
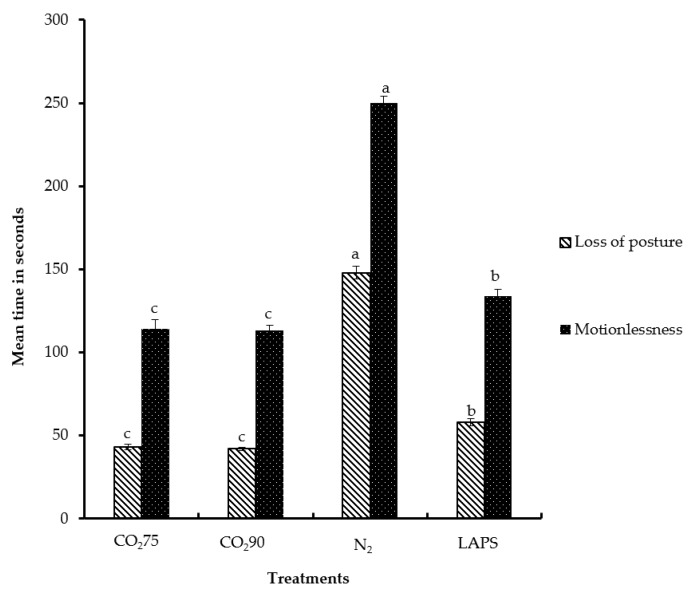
Mean latencies to loss of posture and motionlessness of male layer chicks. The latencies are expressed in s. Bars (mean ± SEM) with different superscripts are significantly different by Fisher’s LSD test (*p* < 0.05). Number of samples per treatment was 10.

Loss of posture is a behavioral indicator of loss of consciousness [44]. It is manifested by the inability to maintain neck tension and body balance [45,46]. In the present study, the latency periods until loss of posture in the chicks subjected to 75% CO_2_, 90% CO_2_, 100% N_2_, and LAPS were 43, 42, 148, and 58 s, respectively (Figure 6). Significant differences were observed in latencies to loss of posture among the four treatments. The chicks subjected to 100% N_2_ took the longest time to lose body posture compared to those in the rest of the treatments (*p* < 0.05). The chicks exposed to the 75% CO_2_ and 90% CO_2_ treatments lost body posture significantly faster than those chicks in the LAPS or 100% N_2_ groups. The chicks subjected to LAPS lost posture earlier than chicks in the 100% N_2_ group, but their loss of posture was later in comparison to those in the CO_2_ inhalation treatments (*p* < 0.05). The findings in this study also demonstrate that CO_2_ is faster than N_2_ in inducing loss of posture in male layer chicks. Poole and Fletcher [46] reported that time to loss of posture in broilers exposed to CO_2_ was significantly shorter than that caused by N_2_. Gerritzen et al. [47] showed that the time to loss of posture of broilers subjected to multistage CO_2_ stunning ranged from 80 to 93 s. Unlike N_2_, exposure to CO_2_ leads to a reduction of intracellular pH [48]. Martoft et al. [7] reported that exposure to higher concentrations of CO_2_ leads to a rapid induction of anesthesia mediated by a decrease in the intracellular pH of the brain. The anesthetic [30] and analgesic effect of CO_2_ [29] may be the probable mechanism for rapid loss of posture. Nitrogen inhalation results in death by anoxia [6]. Unlike CO_2_ inhalation, chicks in the LAPS treatment group did not experience the anesthetic effect. Therefore, chicks in the LAPS method group took a significantly longer time to be affected than those in the CO_2_ treatment groups. Mackie and McKeegan [49] observed that mean latency to loss of posture of broilers subjected to LAPS was 80.7 s.

The cessation of visible movements, including respiratory motion, is a state of motionlessness [25]. The time of latency to motionlessness was determined for chicks in each replicate in all treatment groups except for the negative control group (Figure 6). In this study, chicks exposed to 75% and 90% CO_2_ took 114 and 113 s to reach a state of motionlessness, respectively. The male layer chicks subjected to 100% N_2_ and LAPS were motionless after 250 and 134 s, respectively. The period of latency to motionlessness of the chicks followed a similar pattern to that of latency to loss of posture. The chicks in the 100% N_2_ treatment group took a significantly longer time to become motionless in comparison to those in the rest of the treatment groups. The chicks in the LAPS group took a longer time than those in the 75% CO_2_ and 90% CO_2_ groups, but a shorter time than those in the 100% N_2_ group to become motionless (*p* < 0.05). In the present study, the treatments that induced rapid loss of posture were the ones in which the chicks demonstrated motionlessness faster. Maintenance of an adequate concentration of gas was also vital in preventing any recovery and in achieving death. In the present study, the chicks subjected to the CO_2_ treatments became motionless faster than did those in the 100% N_2_ treatment group. A similar result was reported by Gerritzen et al. [45]. They reported that broilers (two weeks old) exposed to 100% CO_2_ become motionless significantly faster than did those in the other treatment groups, including the 50% N_2_ + 50% CO_2_ and 30% O_2_ + 30% N_2_ + 40% CO_2_ gas mixture treatments. Broilers stunned by LAPS took an average of 199.4 s to become motionless, with a range of 158.2 s to 245.6 s [49]. The latency period to motionlessness of the chicks in this study that were subjected to LAPS ranged from 115 to 151 s.

## 4. Conclusions

In all treatments, chicks experienced noise produced by the flow of gases or operation of the vacuum pump. Chicks exposed to the air treatment were alive for the longest duration among all of the treatments. Noise could have been one of the factors that stressed the chicks in the air group, resulting in higher CORT levels and lower 5-HT levels. This could be one of the limitations of the study, and future studies should probably account for the effects of noise on the stress response. The current research findings demonstrate that the LAPS method can be as effective as CO_2_ or N_2_ inhalation for humane killing of recently hatched male layer chicks. However, the air pressure has to be reduced to 15.3 kPa from 100.12 kPa for chicks to be euthanized using LAPS. The gas inhalation and LAPS treatments are similar in terms of physiological stress responses. However, the use of CO_2_ results in a faster onset of unconsciousness and leads to death earlier than the other methods. This study shows that a CO_2_ concentration of 75% in air is effective for the humane killing of male layer neonates. Similarly, residual oxygen concentration should be maintained at or below 2% if N_2_ gas is being used for male chick euthanasia. Behavioral responses of chicks in CO_2_ treatment prior to loss of posture were different from chicks subjected to N_2_ and LAPS treatments. Future studies should focus on quantifying and assessing such differences.

## References

[1] Conway A. (2016). Egg Production Breaks Record. http://www.poultrytrends.com/2016/#/32.

[2] U.S. Department of Agriculture (USDA), National Agricultural Statistics Service (2017). Chicken and Eggs: 2016 Summary. http://usda.mannlib.cornell.edu/usda/current/ChickEgg/ChickEgg-02-27-2017.pdf.

[3] Ellendorff F., Klein S. (2003). Current knowledge on sex determination and sex diagnosis: Potential solutions. World’s Poult. Sci. J..

[4] Gerken M., Jaenecke D., Kreuzer M. (2003). Growth, behaviour and carcass characteristics of egg-type cockerels compared to male broilers. World’s Poult. Sci. J..

[5] Leenstra F., Munnichs G., Beekman V., Van Den Heuvel-Vromans E., Aramyan L., Woelders H. (2011). Killing day-old chicks? Public opinion regarding potential alternatives. Anim. Welf..

[6] American Veterinary Medical Association (2013). AVMA Guidelines on Euthanasia. https://www.avma.org/KB/Policies/Documents/euthanasia.pdf.

[7] Martoft L., Stødkilde-Jørgensen H., Forslid A., Pedersen H.D., Jørgensen P.F. (2003). CO_2_ induced acute respiratory acidosis and brain tissue intracellular pH: A 31P NMR study in swine. Lab. Anim..

[8] Gerritzen M.A., Lambooij E., Reimert H.G.M., Spruijt B.M., Stegeman J.A. (2006). Susceptibility of duck and turkey to severe hypercapnic hypoxia. Poult. Sci..

[9] Raj A.B.M., Whittington P.E. (1995). Euthanasia of day-old chicks with carbon dioxide and argon. Vet. Rec..

[10] Council of the European Union (1993). Council Directive 93/119/EC of 22 December 1993 on the Protection of Animals at the Time of Slaughter or Killing. https://www.ecolex.org/details/legislation/council-directive-93119ec-on-the-protection-of-animals-at-the-time-of-slaughter-or-killing-lex-faoc018522/.

[11] Webster A.B., Fletcher D.L. (2004). Assessment of the aversion of hens to different gas atmospheres using an approach-avoidance test. Appl. Anim. Behav. Sci..

[12] Raj A.B.M. (2006). Recent developments in stunning and slaughter of poultry. World’s Poult. Sci. J..

[13] American Veterinary Medical Association (2016). Guidelines for the Humane Slaughter of Animals. https://www.avma.org/KB/Resources/Reference/AnimalWelfare/Documents/Humane-Slaughter-Guidelines.pdf.

[14] Vizzier-Thaxton Y., Christensen K.D., Schilling M.W., Buhr R.J., Thaxton J.P. (2010). A new humane method of stunning broilers using low atmospheric pressure. J. Appl. Poult. Res..

[15] Purswell J.L., Thaxton J.P., Branton S.L. (2007). Identifying process variables for a low atmospheric pressure stunning-killing system. J. Appl. Poult. Res..

[16] Berg C., Raj M. (2015). A review of different stunning methods for Poultry-animal welfare aspects (stunning methods for poultry). Animals.

[17] Martin J.E., Christensen K., Vizzier-Thaxton Y., Mitchell M.A., McKeegan D.E.F. (2016). Behavioural, brain and cardiac responses to hypobaric hypoxia in broiler chickens. Physiol. Behav..

[18] Anton F., Euchner I., Handwerker H.O. (1992). Psychophysical examination of pain induced by defined CO_2_ pulses applied to the nasal mucosa. Pain.

[19] Mckeegan D.E.F., Mcintyre J., Demmers T.G.M., Wathes C.M., Jones R.B. (2006). Behavioural responses of broiler chickens during acute exposure to gaseous stimulation. Appl. Anim. Behav. Sci..

[20] Raj A.B.M. (1998). Changes in the somatosensory evoked potentials and spontaneous electroencephalogram of broiler chickens during exposure to gas mixtures. Br. Poult. Sci..

[21] Lambooij E., Gerritzen M.A., Engel B., Hillebrand S.J.W., Lankhaar J., Pieterse C. (1999). Behavioural responses during exposure of broiler chickens to different gas mixtures. Appl. Anim. Behav. Sci..

[22] Jaksch W. (1981). Euthanasia of day-old male chicks in the poultry industry. Int. J. Study Anim. Probl..

[23] Sandilands V., Raj A.B.M., Baker L., Sparks N.H.C. (2011). Aversion of chickens to various lethal gas mixtures. Anim. Welf..

[24] Vizzier Thaxton Y., Christensen K.D., Mench J.A., Rumley E.R., Daugherty C., Feinberg B., Parker M., Siegel P., Scanes C.G. (2016). Animal welfare challenges for today and tomorrow. Poult. Sci..

[25] Coenen A.M.L., Lankhaar J., Lowe J.C., McKeegan D.E.F. (2009). Remote monitoring of electroencephalogram, electrocardiogram, and behavior during controlled atmosphere stunning in broilers: Implications for welfare. Poult. Sci..

[26] Steel R.G.D., Torrie J.H. (1980). Principles and Procedures of Statistics: A Biometrical Approach.

[27] Lucke J.N. (1979). Euthanasia in small animals. Vet. Rec..

[28] Kaye J., Buchanan F., Kendrick A., Johnson P., Lowry C., Bailey J., Nutt D., Lightman S. (2004). Acute carbon dioxide exposure in healthy adults: Evaluation of a novel means of investigating the stress response. J. Neuroendocrinol..

[29] Otsuguro K., Yasutake S., Yamaji Y., Ban M., Ohta T., Ito S. (2008). Why does carbon dioxide produce analgesia?. Proceedings of the 6th World Congress on Alternatives & Animal Use in the Life Sciences.

[30] Brosnan R.J., Eger E.I., Laster M.J., Sonner J.M. (2007). Anesthetic properties of carbon dioxide in the rat. Anesth. Analg..

[31] Berger M., Gray J.A., Roth B.L. (2009). The Expanded Biology of Serotonin. Annu. Rev. Med..

[32] Mohammad-Zadeh L.F., Moses L., Gwaltney-Brant M. (2008). Serotonin: A review. J. Vet. Pharmacol. Therap..

[33] Carrasco G.A., Van De Kar L.D. (2003). Neuroendocrine pharmacology of stress. Eur. J. Pharmacol..

[34] Coppen A., Turner P., Roswell A. (1976). 5-HT in the whole blood of patients with depressive illness. Postgrad. Med. J..

[35] Alshaarawy O., Anthony J.C. (2015). Reduced whole blood serotonin in major depression. Depress. Anxiety.

[36] Le Quan-Bui K.H., Plaisant O., Leboyer M., Gay C., Kamal L., Devynck M.A., Meyer P. (1984). Reduced platelet serotonin in depression. Psychiatry Res..

[37] Williams E., Stewart-Knox B., Helander A., McConville C., Bradbury I., Rowland I. (2006). Associations between whole-blood serotonin and subjective mood in healthy male volunteers. Biol. Psychol..

[38] Uitdehaag K.A., Rodenburg T.B., Van Reenen C.G., Koopmanschap R.E., De Vries Reilingh G., Engel B., Buist W.G., Komen H., Bolhuis J.E. (2011). Effects of genetic origin and social environment on behavioral response to manual restraint and monoamine functioning in laying hens. Poult. Sci..

[39] Rodenburg T.B., Bolhuis J.E., Koopmanschap R.E., Ellen E.D., Decuypere E. (2009). Maternal care and selection for low mortality affect post-stress corticosterone and peripheral serotonin in laying hens. Physiol. Behav..

[40] Bolhuis J.E., Ellen E.D., Van Reenen C.G., De Groot J., Ten Napel J., Koopmanschap R.E., De Vries Reilingh G., Uitdehaag K.A., Kemp B., Rodenburg T.B. (2009). Effects of genetic group selection against mortality on behavior and peripheral serotonin in domestic laying hens with trimmed and intact beaks. Physiol. Behav..

[41] Gurung S., White D., Archer G., Styles D., Zhao D., Farnell Y., Byrd J., Farnell M. (2018). Carbon dioxide and nitrogen infused compressed air foam for depopulation of caged laying hens. Animals.

[42] Inoue T., Koyama T. (1996). Effects of acute and chronic administration of high-dose corticosterone and dexamethasone on regional brain dopamine and serotonin metabolism in rats. Prog. Neuro Psychopharmacol. Biol. Psychiatry.

[43] Beausoleil N.J., Mellor D.J. (2015). Introducing breathlessness as a significant animal welfare issue. N. Z. Vet. J..

[44] Gerritzen M., Lambooij B., Reimert H., Stegeman A., Spruijt B. (2007). A note on behaviour of poultry exposed to increasing carbon dioxide concentrations. Appl. Anim. Behav. Sci..

[45] Gerritzen M.A., Lambooij B., Reimert H., Stegeman A., Spruijt B. (2004). On-farm euthanasia of broiler chickens: Effects of different gas mixtures on behavior and brain activity. Poult. Sci..

[46] Poole G.H., Fletcher D.L. (1995). A comparison of argon, carbon dioxide, and nitrogen in a broiler killing system. Poult. Sci..

[47] Gerritzen M.A., Reimert H.G.M., Hindle V.A., Verhoeven M.T.W., Veerkamp W.B. (2013). Multistage carbon dioxide gas stunning of broilers. Poult. Sci..

[48] Meyer J.S., Gotoh F., Tazaki Y. (1961). CO_2_ Narcosis: An experimental study. Neurology.

[49] Mackie N., McKeegan D.E.F. (2016). Behavioural responses of broiler chickens during low atmospheric pressure stunning. Appl. Anim. Behav. Sci..

